# A simple optical pH sensor based on pectin and *Ruellia tuberosa *L-derived anthocyanin for fish freshness monitoring

**DOI:** 10.12688/f1000research.52836.2

**Published:** 2021-07-21

**Authors:** Nazaruddin Nazaruddin, Nurul Afifah, Muhammad Bahi, Susilawati Susilawati, Nor Diyana Md. Sani, Chakavak Esmaeili, Muhammad Iqhrammullah, Murniana Murniana, Uswatun Hasanah, Eka Safitri

**Affiliations:** 1 Department of Chemistry, Faculty of Mathematics and Natural Sciences, Universitas Syiah Kuala, Banda Aceh, Aceh, 23111, Indonesia; 2 Sanichem Resources Sdn. Bhd., Bandar Estek, Negeri Sembilan, 71060, Malaysia; 3 Center of Excellence in Electrochemistry, University of Tehran, Tehran, 14176-14411, Iran; 4 Graduate School of Mathematics and Applied Sciences, Universitas Syiah Kuala, Banda Aceh, Aceh, 23111, Indonesia; 5 Department of Fisheries, Universitas Teuku Umar, West Aceh, Aceh, 23615, Indonesia

**Keywords:** optical pH sensor, matrix membrane, pectin, anthocyanin, fish freshness

## Abstract

A simple optical pH sensor using the active compound anthocyanin (ACN), derived *Ruellia tuberosa* L. flower immobilized in a pectin membrane matrix, was been fabricated and employed to monitor the freshness of tilapia fish at room temperature and 4 ^o^C storage. The quantitative pH values were measured based on the UV-Vis spectroscopy absorbance. The optimum pectin weight and ACN concentrations were 0.1% and 0.025 mg/L. The sensor showed good sensitivity at 0.03 M phosphate buffer solution. The sensor’s reproducibility was evaluated using 10 replicate sensors where a standard deviation of 0.045 or relative standard deviation of 9.15 was achieved. The sensor displayed an excellent response after 10 minutes of exposure, possessing a response stability for 10 consecutive days. The decrease in pH value of the Tilapia fish from 7.3 to 5 was observed in a 48 hour test, which can be used as the parameter when monitoring fish freshness. Overall, this reported optical pH sensor has a novelty as it could be used to monitor the rigor mortis phase of fish meat, which is useful in food industry.

## Introduction

Fish freshness assessment is the main concern for consumers nowadays as people are more cautious about what they put into their body. Eating spoiled products will cause food poisoning symptoms to various degrees. For example, eating spoiled fish may result in an almost immediate onset of diarrhea, nausea and vomiting. According to the United Nations, about 4.5 billion people rely on fish for 15% of their animal protein intake. ^1^ Therefore, it is imperative to monitor the freshness and quality of fish. Currently, consumers rely on their own experience in determining fish freshness. This is mostly based on the physical condition of the fish like its color and smell. This method is very subjective; hence, there is a need for a more quantitative monitoring method for fish freshness. Heising *et al.* (2012) ^2^ has produced a fish freshness monitoring method by detecting total volatile basic nitrogen using an ammonia ion-selective electrode. However, not all of the ammonia produced will dissociate in the aqueous phase, which is a challenge in the conductivity changes-dependent method. Determination of fish freshness can also be performed by measuring trimethylamine (TMA) levels using electrochemical sensing, as reported by Bourigua *et al.* (2011) ^3^ However, determining the freshness of fish via measuring TMA requires a complicated procedure and experts to operate the equipment. Fish freshness can be monitored using an ammonia optical sensor. Wells *et al.* (2019) ^4^ reported the determination of fish freshness through ammonia measurement that also used TMA solution standard and a dye indicator for pH measurement. Beside these two methods, a pH sensor can also be employed to monitor fish freshness. ^5– 8^ There have been several methods proposed to determine pH levels of a fish sample. The most common methods used are optical sensors and ion-selective electrodes (ISEs). ^9^ The measurement of pH using an H ^+^ ISE is dramatically affected by interferences from samples, especially the presence of alkaline ions. ^10^ Thus, the determination of pH through optics may be an excellent alternative for samples that contain interfering ions.

Several organic pH-sensitive dyes, immobilized in synthetic membranes, have been utilized in the construction of optical pH sensors. Nonetheless, safer compounds derived from natural products have attracted the attention of researchers in developing pH sensors. An earlier report of optical pH sensors includes the construction of a pH sensor using phenol red as an active molecule. ^11^ The further report had described the development of a pH sensor utilizing polyvinyl chloride as the matrix and the fluorescence compound fluorescein-O-methacrylate as the active molecule. ^12^ Nevertheless, these aforementioned pH sensors could only be used on solutions with near-neutral pH as more basic or acidic solutions will give an insignificant response time. Pourjavaher *et al.* (2017) ^13^ has designed a pH sensor using bacterial cellulose (BC) nanofiber matrix to immobilize anthocyanin (CAN) from red cabbage (Brassica oleracea) extract. The sensor has a fairly wide pH range but it needs further characterization to evaluate the sensor performance, especially, for real foodstuff analysis. The use of anthocyanin ACN from blackberries and chitosan membrane in an optical pH sensor has been established. ^14^ The interaction and mechanical properties of chitosan membrane with entrapped ACN have also been reported. ^15^ Anthocyanins are flavonoids possessing a number of hydroxyl groups contributing a strong interaction with chitosan via hydrogen bonding.

A more recent study on fish freshness monitoring through optical methods was reported by Moradi *et al.* (2019) ^16^ using nanofiber bacterial cellulose with ACN. However, this method requires a relatively long analytical time as the pH measurement could not be conducted *in situ.* Chen *et al*. (2020) ^7^ has developed a sensitive novel film prepared from starch polyvinyl alcohol and starch polyvinyl alcohol glycerol. The study used curcumin from turmeric and anthocyanin from purple sweet potatoes. The results showed that the mixture of curcumin and ACN improved the stability than that of the individual active substances. As the consequence, the sensor could be employed to detect volatile ammonia as the fish freshness indicator.

Herein, we constructed a new optical pH sensor based on pectin (PC) matrix and ACN extract from the *Ruellia tuberosa* L flower. The ACN derived from the crude extract of *Ruellia tuberosa* L flower has been reported to be pH sensitive. ^17^ PC is a non-toxic biopolymer that can be crosslinked with the assistance of CaCl _2_. PC membrane is transparent, deeming it suitable as a matrix for optical measurements. Moreover, PC is also a hydrogel that will enable easy diffusion of analytes leading to a faster response time compared to another hydrophobic matrices. ^18^ In addition, PC application as an optical pH sensor for fish freshness monitoring has not been well-explored. ACN is well known to be pH sensitive and will undergo color changes at different pH. ^19^ This compound is easily obtained from nature and is relatively cheap compared to other pH sensitive active molecules. In the present work, ACN has been extracted from the flower *Ruellia-tuberosa* L. The ACN was immobilized onto PC membrane to produce CAN/PC composite membrane which can be used for *in situ* detection of fish freshness without requiring a destructive procedure.

## Methods

### Materials

All chemicals used in this research are analytical grade. Monopotassium phosphate (KH _2_PO _4_) and dipotassium phosphate (K _2_HPO _4_) were purchased from Merck (Merck Millipore, Darmstadt, Germany); PC, ethanol, and CaCl _2_ – from Sigma-Aldrich (Sigma Aldrich Chemie GmbH, München, Germany); and methanol and acetic acid – from Fluka (Fluka Chemie GmbH, Buchs, Switzerland). As for the plant sample, wild *Ruellia tuberosa* L. was collected from the area near Universitas Syiah Kuala in Banda Aceh, Aceh, Indonesia. To study the application of the optical pH sensor on the real sample, dead tilapia fishes were used and purchased from the traditional market in Banda Aceh, Aceh, Indonesia.

### Study design

**Figure 1.**
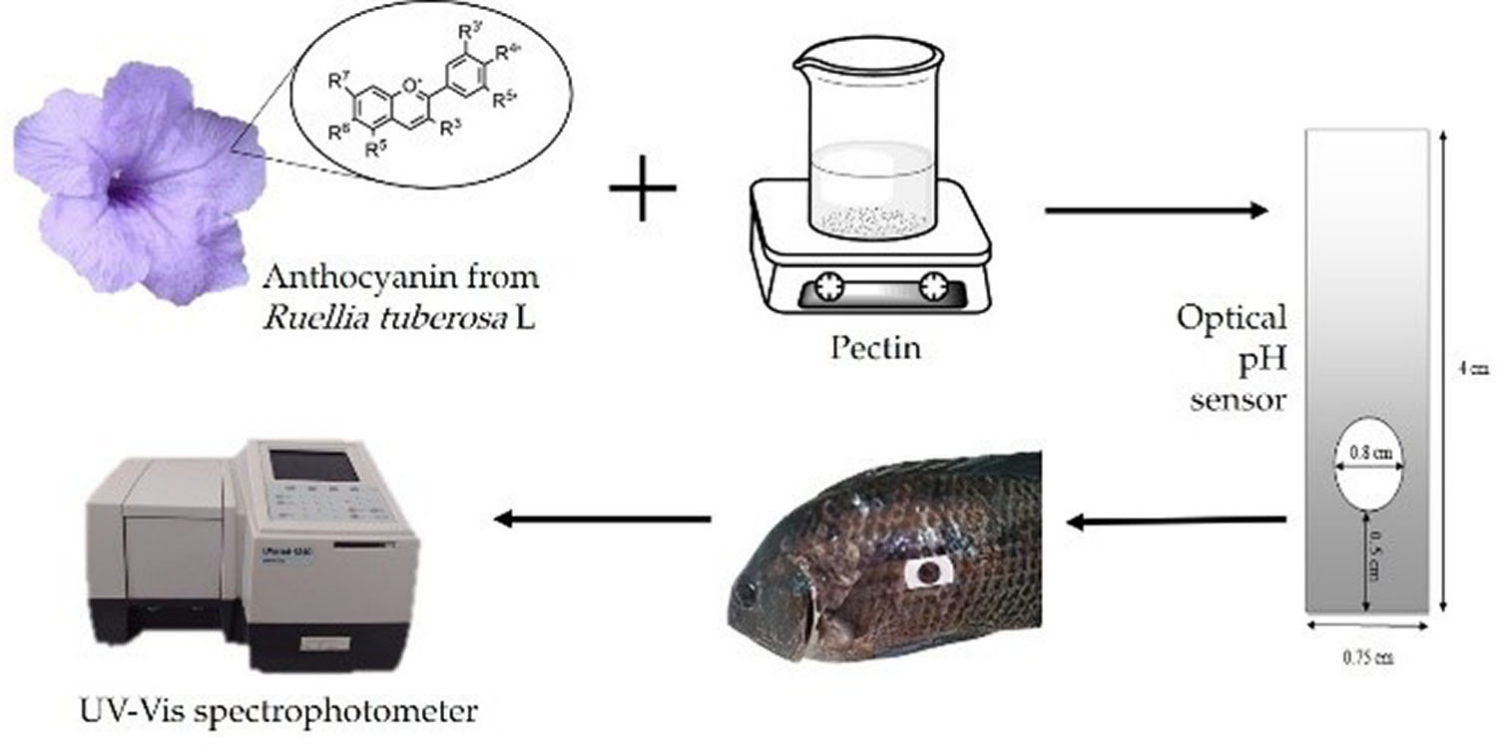
Schematic diagram of optical sensor fabrication and its application for fish freshness monitoring.

The first step in sensor fabrication was the extraction of anthocyanin from *Ruellia tuberosa* L. The extracted anthocyanins were then mixed with pectin solution and printed proportionally as an optical pH sensor. The optical pH sensor was then characterized and the optimized and then applied to monitor the freshness of tilapia. The image below is a schematic diagram summarizing research procedures conducted in this work ( Figure 1).

### Anthocyanin extraction

The procedure follows a previous report. ^20^ Briefly, 200 g fresh *R. tuberosa* L. was macerated in 85 mL methanol for 24 h at room temperature (32-34°C). The residue was then separated from the filtrate by simple filtration. Finally, ACN was obtained after the solvent was removed from the filtrate by means of steaming at 50°C until the volume reached 50 mL.

### Construction of optical pH sensor with various ACN concentrations

**Figure 2.**
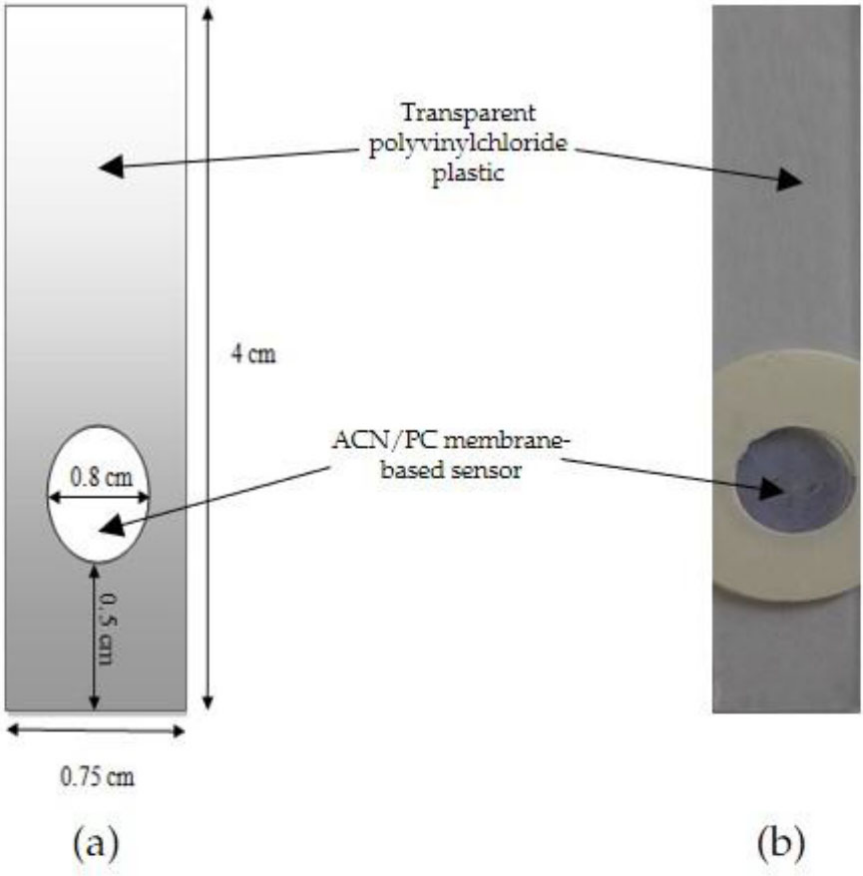
(a) The designed shape and (b) the visual appearance of ACN/PC optical pH sensor.

The optical pH sensor was constructed by dissolving PC powder into a matrix solution (0.1% w/v) in 100 mL CaCl _2_ 0.1 M solution, heated at 60°C. After the mixture was cooled down, the previously obtained ACN extract (1.503 mg/L) was added to 1.66, 2.49 and 3.33 mL PC matrix solution to produce three different 100 mL ACN/PC solutions with respective ACN concentrations of 0.025, 0.0375 and 0.05 mg/L. A total of 40 μL the ACN/PC solution was dropped onto a polyvinylchloride plastic mold surface with a diameter of 0.8 cm ( Figure 2). The sensor was allowed to dry for 24 h at 4 ^o^C.

Fourier Transform Infrared (FTIR) Cary 630 Anti Agilent (Penang, Malaysia) was used to identify the structure and functional groups. The membrane morphology was observed under Zeiss Merlin/Merlin Compact/Supra 55VP Field Emission Scanning Electron (FESEM) (Selangor, Malaysia). Thermal stability of the constructed membrane was analyzed using Shimadzu DTG-60 Thermal Gravimetric Analyzer (Kyoto, Japan) and Differential Scanning Calorimetry (DSC) Shimadzu DSC-60 (Kyoto, Japan). Unless otherwise stated, the conditions for these characterizations followed that of reported work for film specimens. ^21, 22^

To test its response and evaluate its analytical performance, each sensor was dripped with 30 μL 0.1 M phosphate buffer solution with a variety of pH values ranging from 5.0 to 8.5 with 0.5 interval –the pH values of each phosphate solution on the sensor were checked by pH-meter Thermo Orion Star A2111 (Selangor, Malaysia). The sensor color changed corresponding to the different pH values of the administered buffer solutions. It consequently resulted in the difference of the absorbance that was then measured nm using UV-VIS Spectrophotometer (Shimadzu Uv-mini-1240, Kyoto, Japan) at λ _max_ = 635, ^17^ until the sensitivity value for pH determination was obtained.

### Effect of PC concentration

The effect of PC concentration was tested based on % weight of PC in CaCl _2_ 0.1 M solution; 0.05, 0.10, and 0.15%. In total, 40 μL of the three different PC solutions containing 0.025 mg/L ACN were casted as previously explained above. Finally, the pH sensor was pipetted with 30 μL phosphate buffer 0.1 M (pH 4-9), and its absorbance was measured.

### Selection of the optimum buffer solution and concentration

The optical pH sensor with optimum ACN and PC concentrations was used to test its performance against phosphate and citrate buffers 0.1 M (pH 5.0-8.5) to select which buffer generated the best outcome. To select the optimum buffer concentration (once the best buffer had been chosen; phosphate), the best buffer solution was varied in concentration (0.01, 0.03, and 0.05 M) and used in the optical pH sensor performance with pH ranging from 6-8 following the previously explained procedure. The optimum concentration was selected based on its sensitivity and linearity of the absorbance versus pH plotting curve.

### Evaluation of reproducibility, response time and lifetime study of the optical pH sensor

Response time of the optical pH sensor was determined by measuring the optimum absorbance of the pH sensor at a range of 5, 10, 15, 20, 25 and 30 minutes. For reproducibility, the performance was conducted 10 times using ten optical pH sensors. For the determination of the optical pH sensor’s lifetime, the absorbance measurement was carried out after 1, 2, 3, 4, 5, 10, 15 and 20 days after the sensor preparation. All of these studies were conducted under optimum buffer conditions.

### Optical pH sensor test on fish sample

The pH values of the tilapia fishes were measured by attaching the sensors onto the fishes' surface for 5 minutes before measuring the absorbance, as explained before. The fish were stored at 4°C and ambient temperature (32-34°C). The pH analysis was carried out every 7, 12, 24, and 48 h of the storage time.

## Results and discussion

### Characteristics: structure, crystallinity, morphology, and thermal behavior

**Figure 3.**
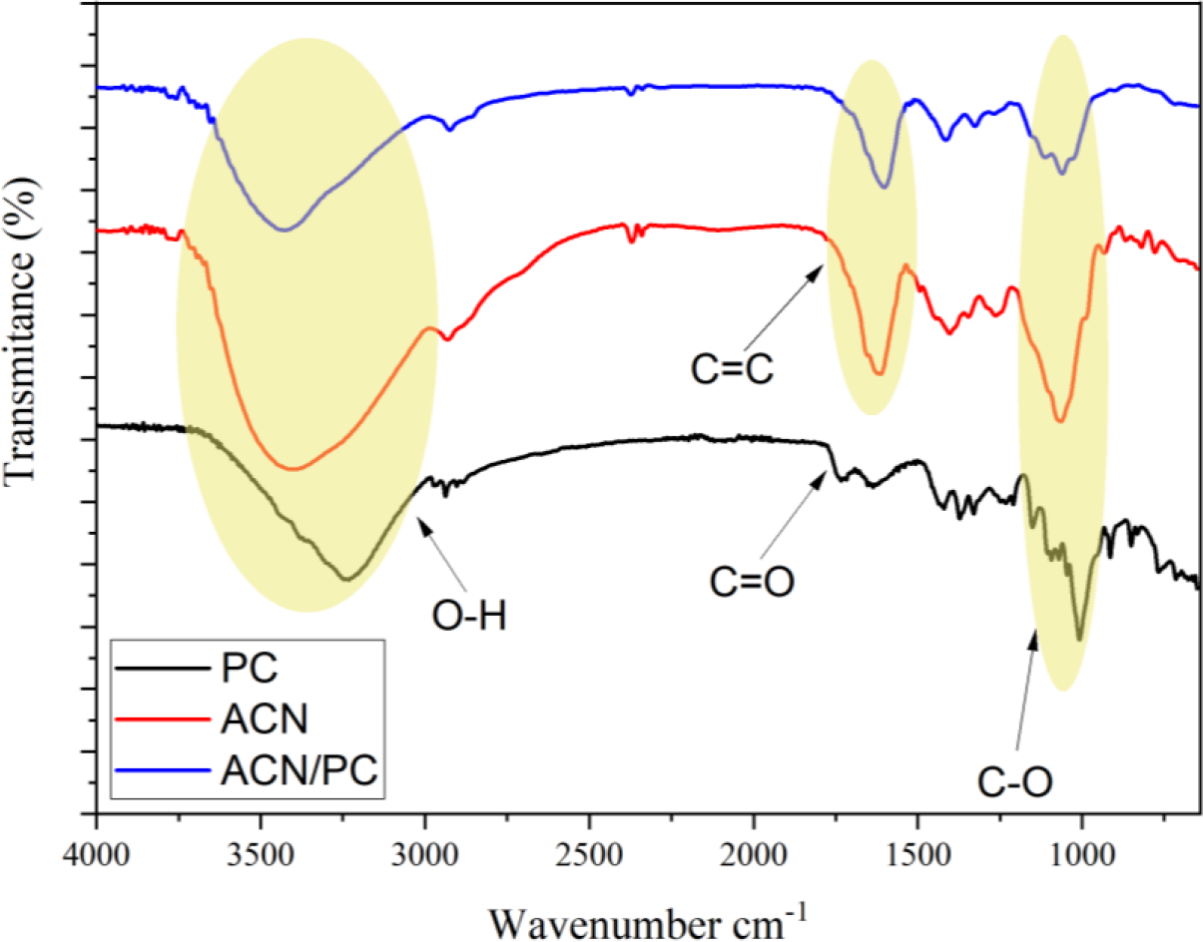
FT-IR spectral profile of PC, ACN, and ACN/PC.

Anthocyanin (ACN) is one of the most important components in the construction of this optical pH sensor other than PC. ACN is obtained from the extract of *R. tuberosa* L. flower that displays different colors at different acidic or basic pH levels. ^23, 24^ FTIR analysis of the extract showed that the broadening vibrational band with medium intensity at the wavenumber, ranged between 3333 cm ^-1^ and 3291 cm ^-1^, indicating the presence of free O-H groups ( Figure 3). The presence of the aromatic C=C vibrations at wavelength region 1644 cm ^-1^ and 1454 cm ^-1^ indicates the typical characteristics of an ACN compound. ^25^ The vibrations by group C-O were recognized from wavelength range 1111 and 1015 cm ^-1^. The FT-IR characterization shows that the ACN is in the form of cyanidin-3-glucoside; similar vibration patterns has been reported previously. ^26^

FT-IR characterization on PC displayed typical PC functional groups at wavenumber range of 1000-2000 cm ^-1^. Spectral band at 1717 cm ^-1^ and 1624 cm ^-1^ are assigned to be vibrations of C=O stretching from ester and carboxylate. The presence of other spectral band at 3370 cm ^-1^ is assigned to the vibrational absorbance of O–H functional groups. The ether bonds of C–O–C is observed by the presence of the absorbance peaks at 1219 and 1096 cm ^-1^. In the case of ACN/PC, free O–H groups from the PC molecule were observed from the overlapping band at 3200-3650 cm ^-1^. The other spectral bands at 1630 – 1850 cm ^-1^ and 1050 – 1260 cm ^-1^ are assigned to carbonyl groups (C=O) and symmetrical ether groups (C–O–C) from glycoside bonds, respectively. ^27, 28^

*TGA/DTGA and DSC profiles of PC membrane*

**Figure 4.**
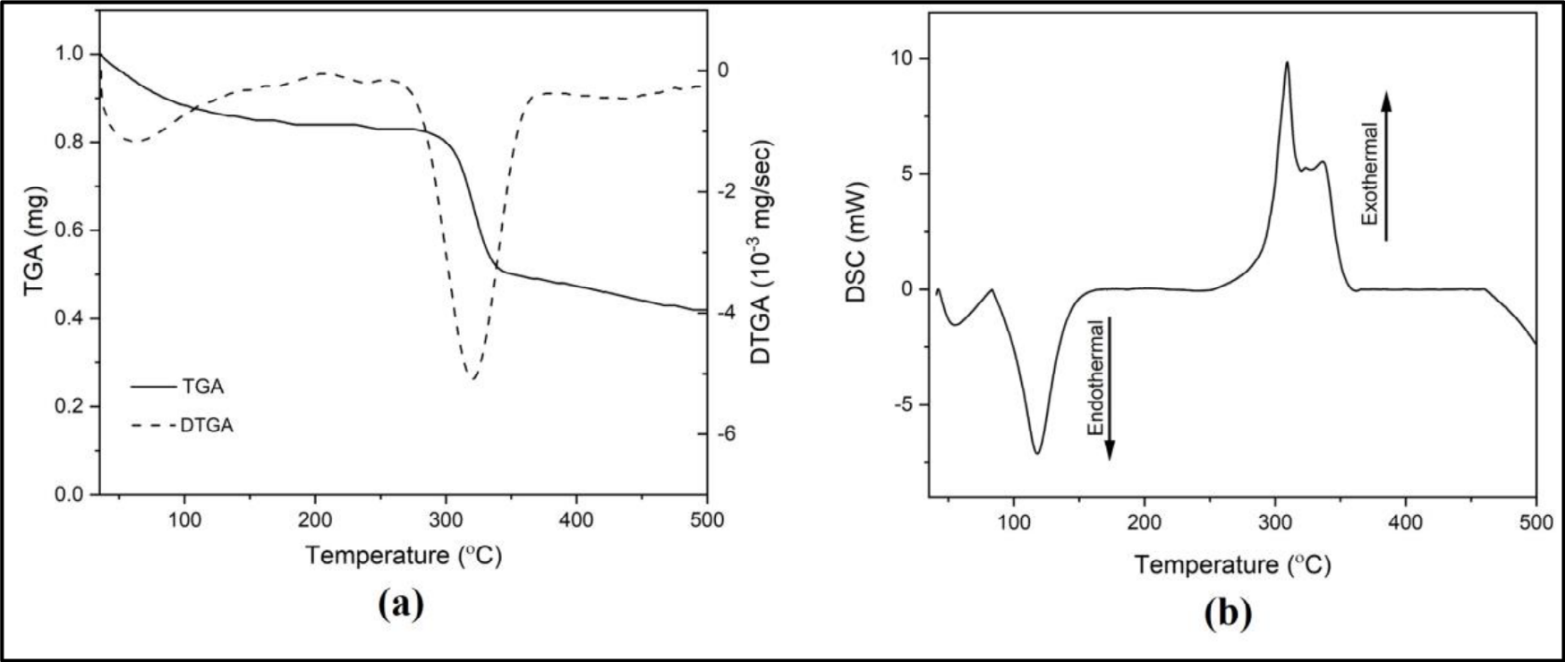
(a) TGA/DTGA and (b) DSC thermograms of thermal analysis on PC membrane.

Thermal stability is one of preferable characteristics when it comes to a bio-sensor as it may influence its performance. We conducted thermal gravimetry analysis (TGA) and differential scanning calorimetry (DSC) studies to assess whether the PC membrane has ideal thermal stability. The thermograms of TGA and its derivative (DTGA) and DSC have been presented in Figure 4a and b. At around 58°C, the release of solvent (water) was observed on the TGA and DTGA thermograms ( Figure 4a). The second peak of DTGA suggests thermal degradation with 30% weight loss. ^29^ A better insight regarding the thermal stability of the PC membrane can be seen in the DSC thermogram. ^21, 22^

The first endothermic peak that appears in the DSC thermogram ( Figure 4b) agrees with the water content release observed in the TGA. T- _onset_ = 83°C indicates the first observable thermal transition, in which it is assigned to melting temperature. It is because within the temperature range (83-118°C), the decrease in weight does not occur in the TGA thermogram. This finding is in line with a previous report investigating PC powder. ^30^ The exothermal peak (T- _peak_ = ± 309°C) observed afterward indicates the degradation of the PC polymeric chain. From these data, we can conclude that the PC membrane is thermally stable at room temperature range.

*SEM images of PC membrane*

**Figure 5.**
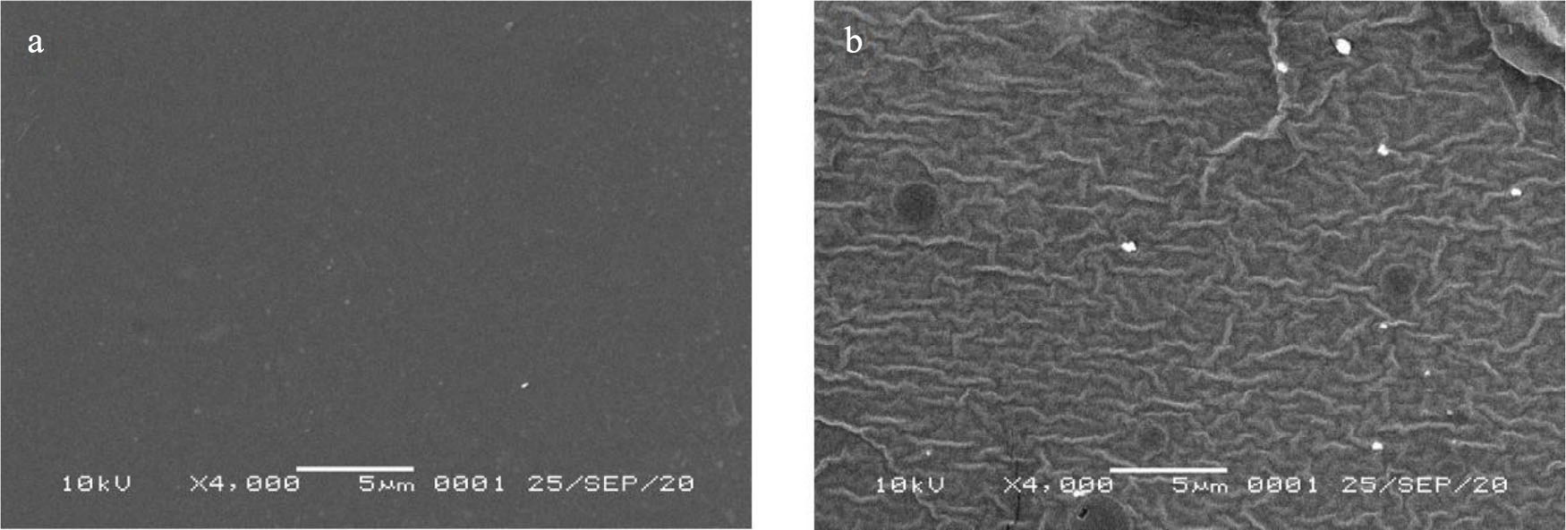
SEM profile of (a) PC and (b) ACN/PC membranes.

SEM images of PC ( Figure 5a) and ACN/PC ( Figure 5b) depict a clear difference of surface morphology between the two. ^18^ PC surface has a morphology that is uniform and smooth. With the addition of ACN into the membrane, wavy layers are shown as the result of the presence of the liquid that, as the consequence, possibly creates a stress tension or air gap. Other study showing severe cracks on the membrane surface, associated with the presence of water. ^31^ This change may lead to poorer sensor performance as a transparent membrane is preferred for optical sensor to allow the UV light passing through the membrane. Hence, investigation on the sensitivity of the optical pH sensor with respect of ACN or PC loads is important.

### Effect of ACN concentration on the sensitivity of the optical pH sensor

**Figure 6.**
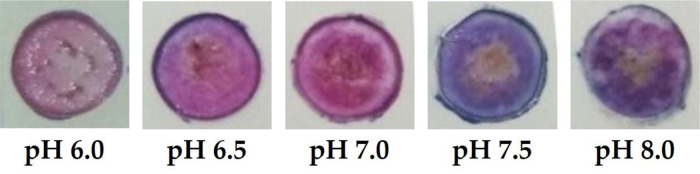
Optical pH sensor color changes at different pH values.

The constructed optical pH sensor based on the ACN derived from *R. tuberosa* L flower has hydrogel characteristics. The advantage of a hydrogel membrane in an optical system is the quick interaction between analyte and active membrane which in turn will accelerate the response time. ^18, 32^ The PC membrane with the immobilized ACN is transparent, where the color change is sensitive against the pH value ( Figure 6). This optical pH sensor is optimized by means of ACN variation to achieve the best sensitivity, observed by a wide linear range and good linearity. Further characterization is followed by the determination of sensor performance.

**Figure 7.**
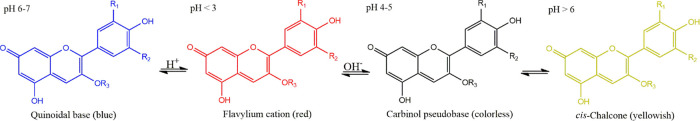
Anthocyanin molecular structures with respect of pH changes.

Color change of ACN can be affected by several factors such as temperature, pH, light intensity, sugar moiety and different phenolic derivatives. ^18^ Due to its solubility in aqueous solution, the color change of ACN is caused by structural transformations of carbon skeleton affected by the levels of H ^+^. Four major anthocyanin skeletons have been reported in the literature at different pH values ( Figure 7); the red flavylium cation (pH < 3), the blue quinoidal base (pH 6-7), the colorless carbinol pseudo-base (pH 4-5), and the yellowish *cis*-chalcone (pH > 6) ( Figure 7). ^33, 34^

**Figure 8.**
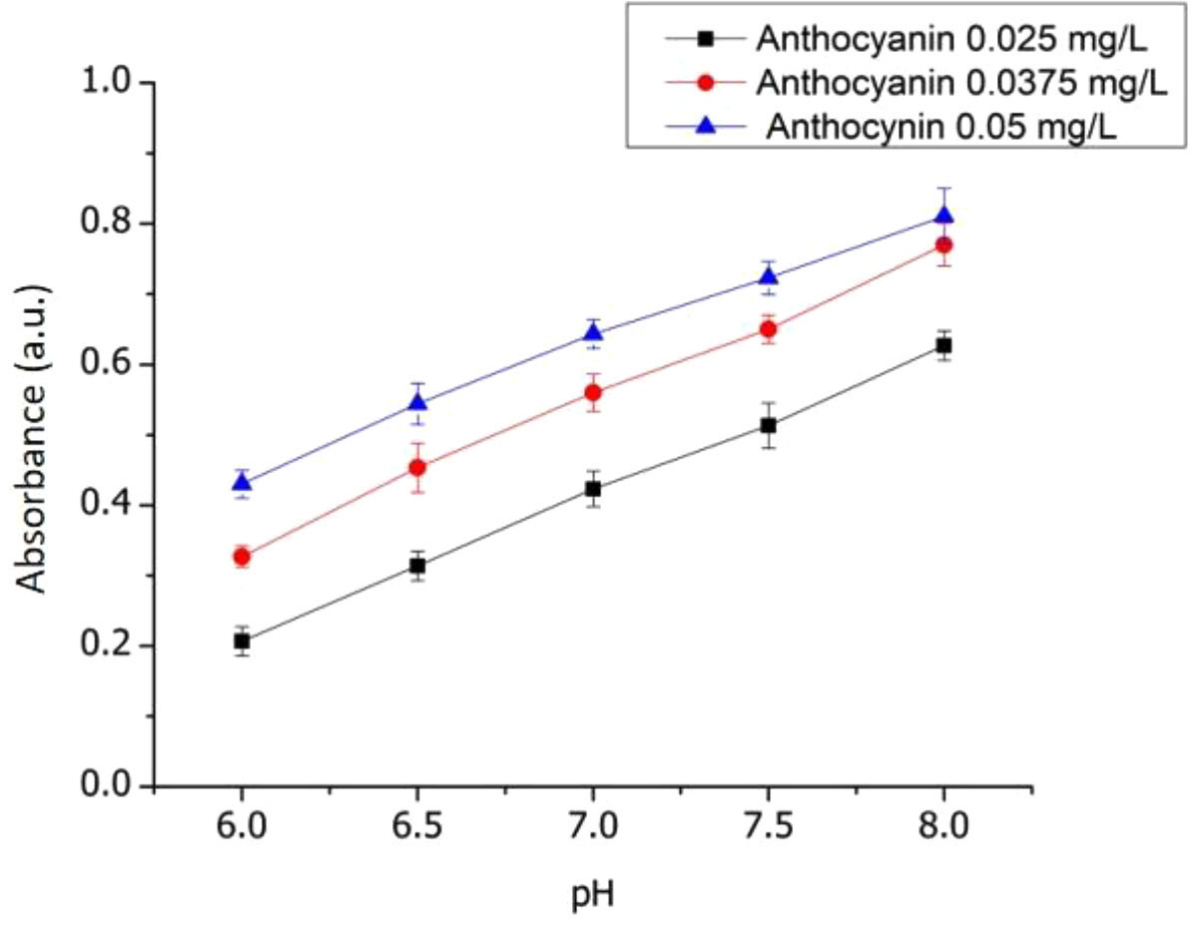
Effect of ACN concentration on sensitivity optical pH sensor.

The effect of ACN concentrations on optical pH sensors response has also been studied and shown ( Table 1 and Figure 8). The sensitivity of the sensor toward variations in ACN concentrations showed not significantly different, but the absorbance vs pH plot showed an increase in the value of the intercept. This indicates the intensity of the sensor color increases with increasing ACN concentrations. Furthermore, the ACN concentration of 0.025 mg/L will be used to construct the optical pH sensor for the next characterization.

**Table 1.** Effect of ACN concentrations on the sensitivity of the optical pH sensors on phosphate buffer.

Concentration (mg/L)	pH range	Sensitivity	R ^2^
0.025	6-8	0.14 ± 0.03	0.999
0.0375	6-8	0.108 ± 0.05	0.999
0.05	6-8	0.094 ± 0.01	0.995

*Effect of PC weight towards **s**ensor **s**ensitivity*

**Figure 9.**
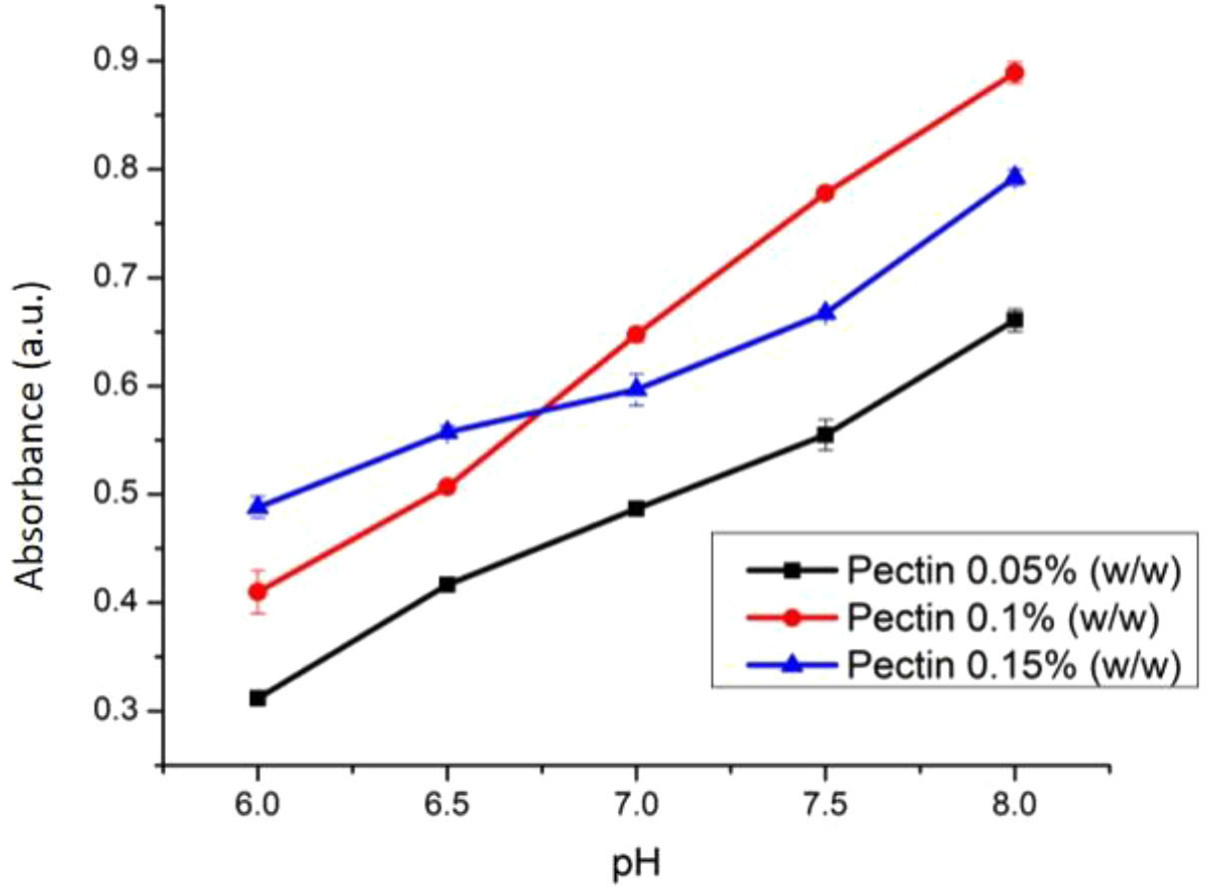
Effect of pectin weight towards the sensitivity of optical pH sensor.

The weight variation of PC (0.05, 0.1, and 0.15% w/v) was studied to find the best sensor sensitivity. At varied weights, PC was dissolved using CaCl _2_ 0.1 M to construct cross linking between Ca ^2+ ion^ and galacturonate until a pectin solution in the form of gel was produced. ^35^ The effect of PC weight towards the sensitivity of optical pH sensor has been presented ( Figure 9). The optimal weight percentage of PC was found at 0.1 % w/v. The membrane with 0.1% w/v pectin has a flatter surface thus making it as the most suitable optical sensor. PC membrane with only 0.05% w/v PC possessed a gel like texture due to the excess of water which causes a longer time to form a solid membrane. This phenomenon is quite similar for membrane preparation using a phase inversion method. ^21, 36, 37^ On the other hand, membrane with 0.15% PC is very dense and has a non-homogenous surface which is not preferred for optical pH membrane application. ^38^

### Effect of type and concentration of buffer on the sensor performance

**Figure 10.**
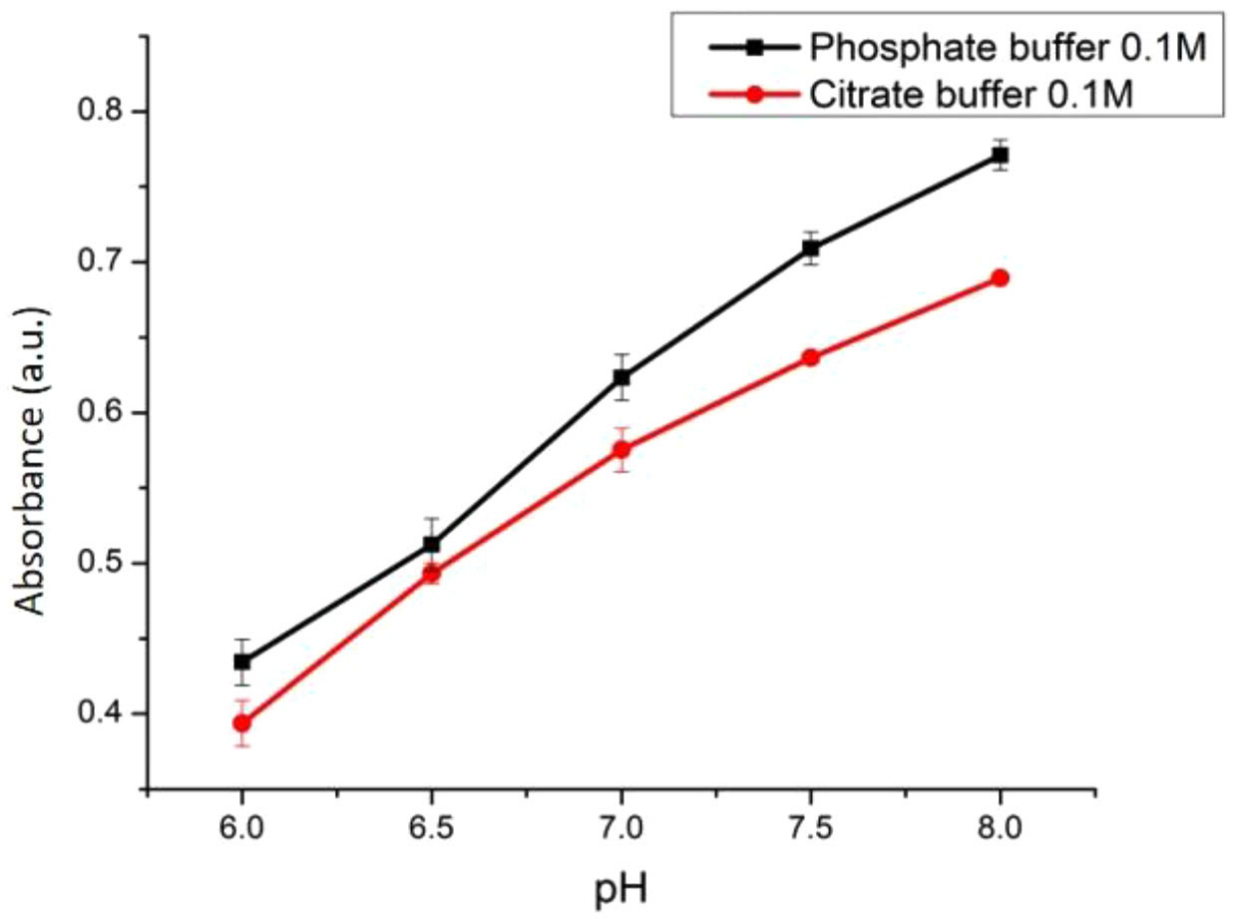
Effect of buffer type towards the sensitivity of optical pH sensor.

The performance of an optical pH sensor may be affected by the types and concentration of the buffer. Figure 10 shows that the sensitivity of the sensor with phosphate buffer was 0.0877 with an R-square value of 0.993. On the other hand, the ACN/PC sensor with citrate buffer had a sensitivity of 0.074 (R ^2^ = 0.981). Through physical observation, the ANC in the sensor would display a higher color intensity when in phosphate buffer compared to citrate buffer even in the same pH range. This is due to the lower K _a_ value of phosphate buffer compared to citrate buffer. Altogether, we conclude that the phosphate buffer contributes to better sensitivity of our pH sensor as opposed to citrate buffer. Therefore, the effect of concentration was studied using the phosphate buffer.

**Figure 11.**
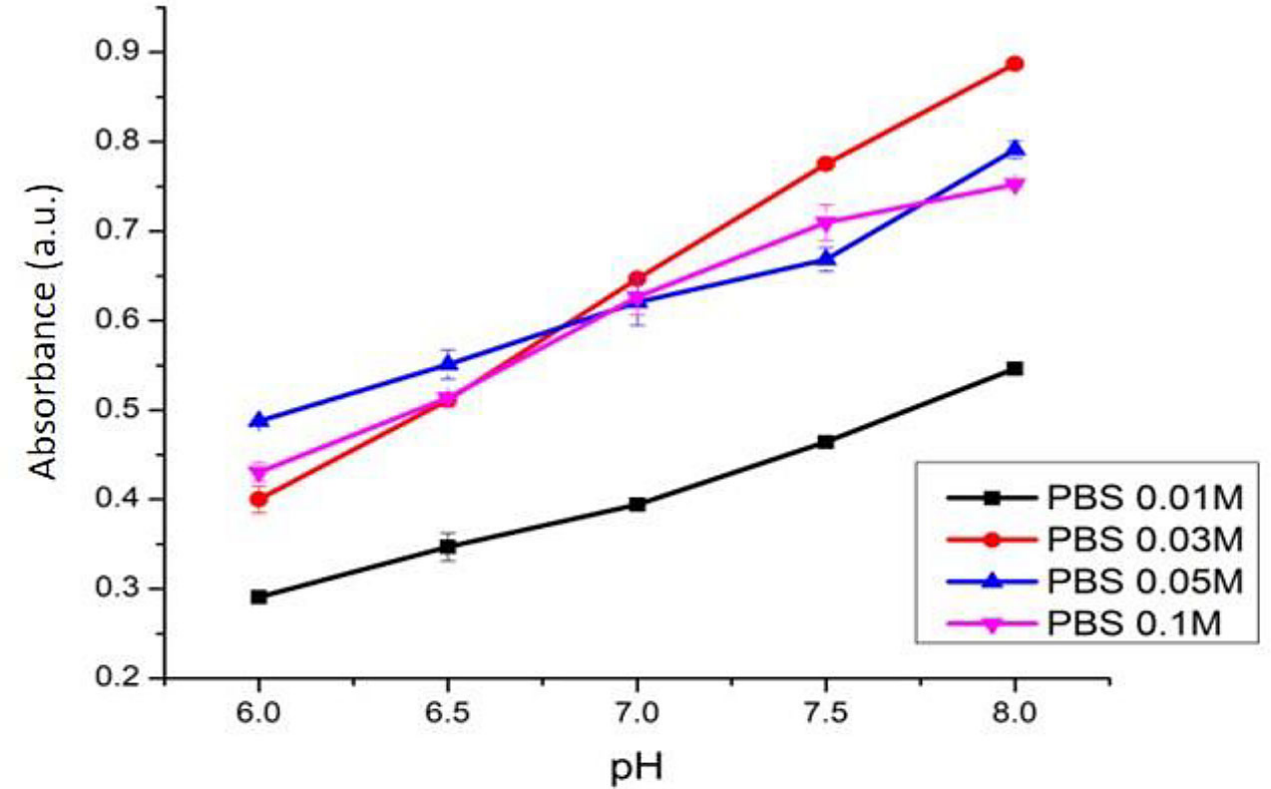
Effect of phosphate buffer concentration towards pH sensor’s sensitivity.

The effect of phosphate buffer concentration towards this sensor’s sensitivity is shown in Figure 11. This pH sensor produces the best sensitivity of 0.1238 (R ^2^ = 0.9989) when the phosphate buffer 0.03 M was used. Meanwhile, the sensitivities of the pH sensor using phosphate buffer with concentrations of 0.05 M and 0.1 M were found lower at 0.072 (R ^2^ = 0.9745) and 0.084 (R ^2^ = 0.9805), respectively. The pH sensor with phosphate buffer 0.03 M gave a more contrast in the color change at different pH levels, in comparison with that of citrate buffer. In comparison to other earlier studies, ^11, 12^ our ACN/PC optical pH sensor has a wider working range of pH.

### Response time and reproducibility measurement

**Figure 12.**
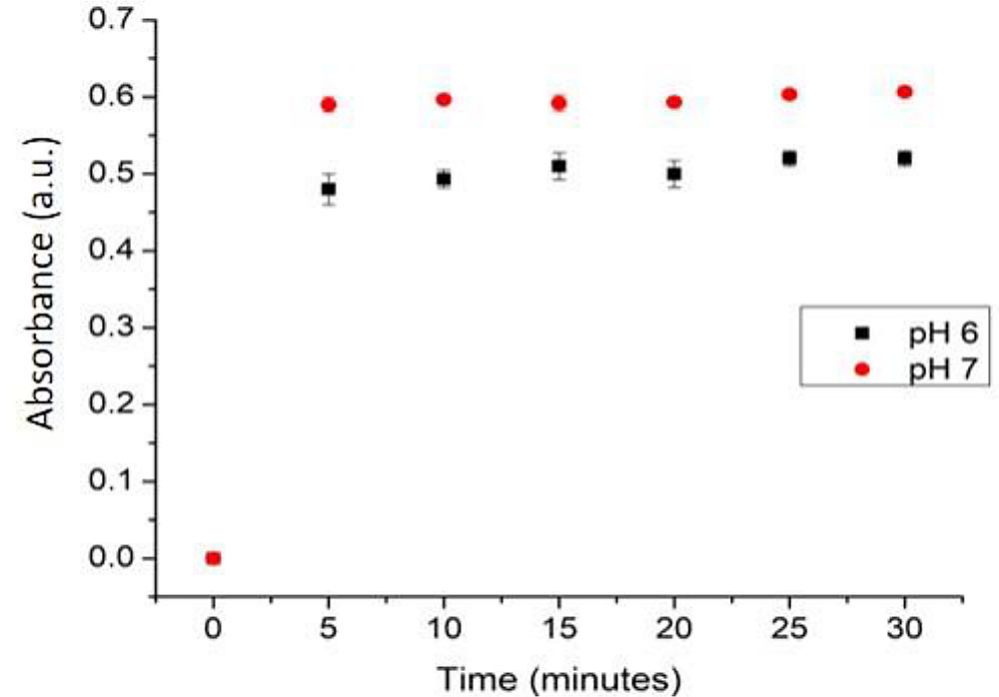
The response time profile of pH sensor.

The response time of this sensor was determined by the required duration (minutes) that the sensor achieves a stable result. Response time was determined at 0, 5, 10, 15, 20, 25, and 30 minutes ( Figure 12). The absorbance increased drastically from the first 5 minutes, indicating a good diffusion of the sample onto the membrane. The increase was later observed at minute 10, but no observable significant change afterward. Therefore, the optimum response time of this optical pH sensor is 10 minutes.

In addition, the reproducibility measurement was conducted on 10 different sensors with the same condition, where the relative standard deviation (RSD) was 9.15. This shows that there is a small difference in the absorbance values obtained from the repetition using new sensors. However, RSD that is below 10% is still acceptable for qualitative measurement. ^38^

### Lifetime of pH sensor

**Figure 13.**
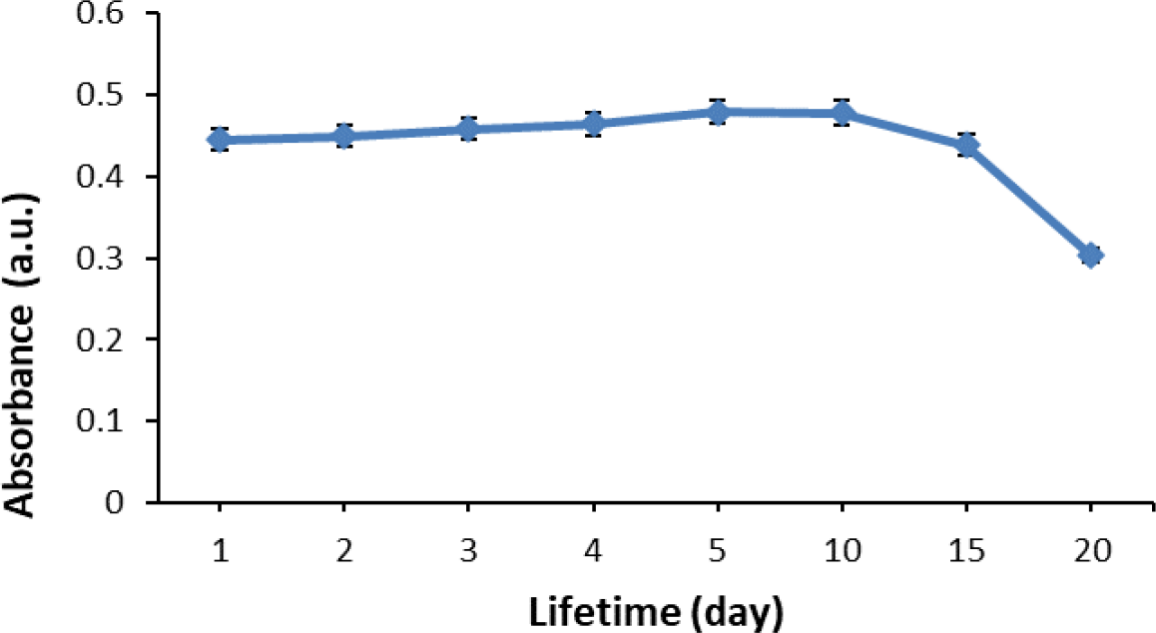
Lifetime of optical pH sensor.

The investigated optical pH sensor had a stable response until the tenth day of storage ( Figure 13). Afterward, the sensor response fell as much as 8.3% from the initial response, in which further decline was observed on the 15 ^th^ day. At the same time, the %RSD also become poor; increasing as much as 36.61% from its initial state. The decrease in sensor performance after particular days of storing depends on the stability of the anthocyanin in maintaining its color. The lifetime of the optical pH sensor in this study is worse in comparison to that of our previous study, ^18^ in which the performance did not drop until the 15 ^th^ day. However, previously we used the synthetic chromoionophore ETH 5294 (CI); unlike in this study where we used natural anthocyanin that can be considered more sustainable. Furthermore, in this study, the lifetime is better in comparison to our currently reported sensor using ACN from *Dioscorea alata* L. ^38^

### Fish freshness test using real samples

**Figure 14.**
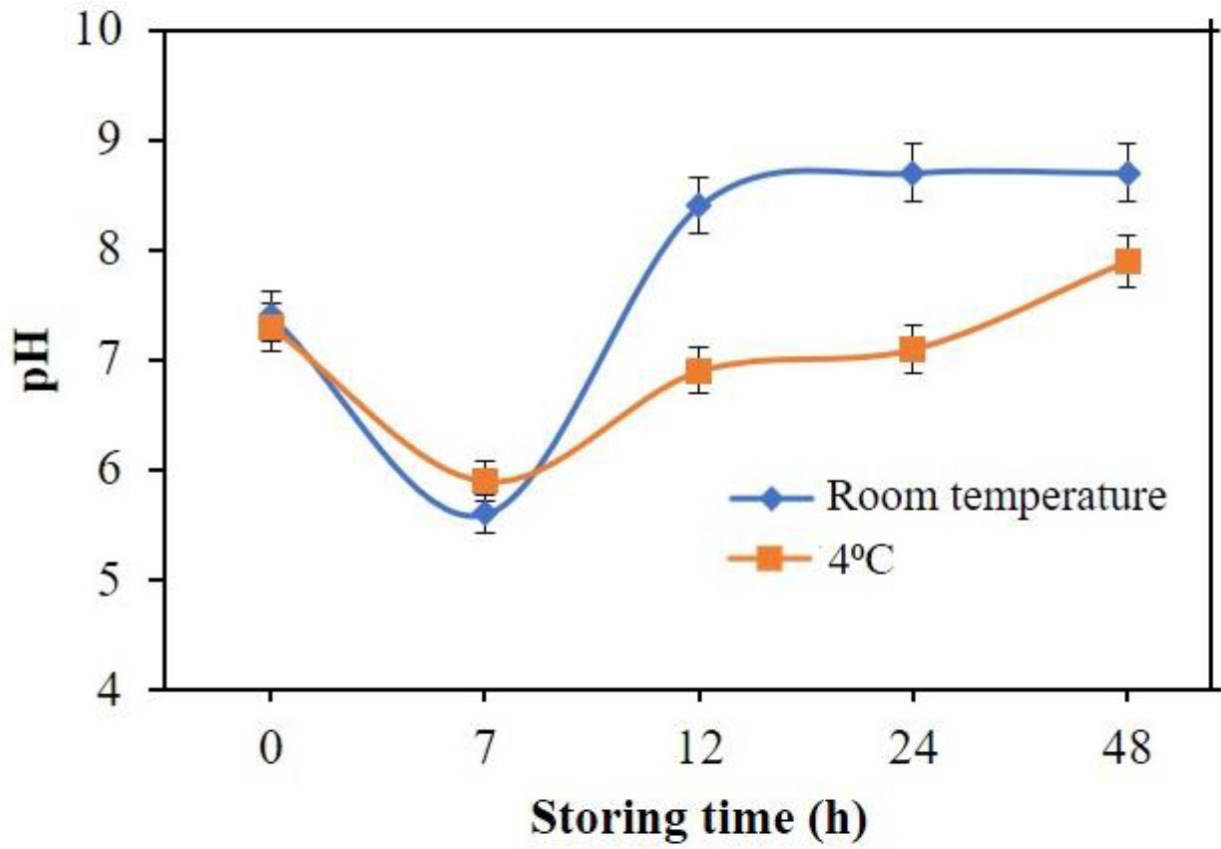
Fish freshness monitoring using optical pH sensor.

Optical pH sensor with the optimal conditions was used to monitor the freshness of tilapia fish that was kept at 4 ^o^C. The pH profile of the fish at two conditions, namely room temperature and 4°C storage temperature, is shown in Figure 14. A living fish has a pH value of around 7.4, but after death the pH decreases. ^39^ The pH of the fish samples was measured after 0, 7, 12, 24 and 48 h storage time at room temperature and 4°C. Fish freshness was measured based on the absorbance value that is converted to pH value based on the constructed calibration curve.

Fish samples kept at room temperature possess a higher pH compared to the fish sample stored at 4°C. Fresh fish that was measured at 0 hours displayed pH of around 7.3-7.4. Following that, the pH decreases to 5.5-5.9, indicating that the fish has reached rigor mortis or postmortem rigidity. The decrease is attributed to the accumulation of lactic acid from post mortem glycolysis. After the rigor mortis phase, the fish will undergo putrefaction due to the microbial activity in the fish sample. ^40^ This activity causes the pH to become more basic due to the breakdown of proteins in the fish sample to become ammonia and trimethylamine. ^23– 25^ Results achieved from pH measurements at 7, 12, 24 and 48 hours at 4°C using the optical sensor yielded results of pH 5.9, 6.9, 7.1 and 7.9. Based on these results, it can be said that fish that is kept at room temperature will undergo a faster decomposition. This is due to the exposure to sunlight thus a higher temperature that will accelerate the process of decomposition.

Our method of measuring the change of pH is different to the most reported studies using colorimetric response. ^4^^,^^5^^,^^7^^,^^14^^,^^16^ Indeed, one may argue that colorimetry could give the best practicality of the sensor use. However, it suffers from quantitative information, as it depends on the RGB profiles that requires complex model to convert the response into measured pH value. Moreover, the reported studies rely on the volatile basic compounds released from the meat. Taken altogether, the reported studies were unable to capture the decrease of pH during rigor mortis phase. In food industry, fish meat is best processed by the filleting machine during the pre- or post-rigor mortem. This is the novelty of our optical pH sensor which is useful for the quality control and processing of fish meat in industrial settings.

## Conclusion

ACN extracted from *Ruellia tuberosa* L can be immobilized into a PC matrix to produce a sensitive optical pH sensor. The extracted ACN has a similarity over the FT-IR profile of cyanidin-3-glucoside. The amount of ACN and PC in the membrane composite affected the optical pH performance, which was largely indicated by intercept and linearity values. The constructed optical pH sensor works best in phosphate buffer with a long lifetime. Its application in monitoring the freshness of fish has been successfully conducted against the storing time, where the decrease in pH values during rigor mortis period were observed. More studies indeed need carried out to obtain smooth surface morphology to improve the optical sensor performance.

## Data availability

### Underlying data

Harvard Dataverse: Data Set for Optical pH Sensor Based on Pectin and Ruellia tuberosa L-derived Anthocyanin for Fish Freshness Monitoring, https://doi.org/10.7910/DVN/ZYCXAM. ^40^

Data are available under the terms of the Creative Commons Zero “No rights reserved” data waiver (CC0 1.0 Public domain dedication).
