# Supplementary material for: A simple optical pH sensor based on pectin and Ruellia tuberosa L-derived anthocyanin for fish freshness monitoring
Source: F1000Res. 2021 Jul 21;10:422. Originally published 2021 May 27. [Version 2] doi: 10.12688/f1000research.52836.2 (PMC8366298; doi:10.12688/f1000research.52836.2)
Supplement: Peer review history

# Peer review: Reviewer response for version 2

**DOI:** 10.5256/f1000research.58582.r90077

**Published:** 2021-08-13

**Authors:** Sagir Alva

**Affiliations:** 1 Department of Mechanical Engineering, Faculty of Engineering, Universitas Mercu Buana, Jakarta, Indonesia

After I read the revised results of this article, the authors of this article has added some suggestions that I have conveyed before, so I think the value of this article is better than before.

Is the work clearly and accurately presented and does it cite the current literature?

Partly

If applicable, is the statistical analysis and its interpretation appropriate?

Yes

Are all the source data underlying the results available to ensure full reproducibility?

Yes

Is the study design appropriate and is the work technically sound?

No

Are the conclusions drawn adequately supported by the results?

Yes

Are sufficient details of methods and analysis provided to allow replication by others?

Yes

Reviewer Expertise:

My specialty is the synthesis and characterization of materials such as polymers for the development of chemical sensors/biosensors.

I confirm that I have read this submission and believe that I have an appropriate level of expertise to confirm that it is of an acceptable scientific standard.

# Peer review: Reviewer response for version 2

**DOI:** 10.5256/f1000research.58582.r90078

**Published:** 2021-08-11

**Authors:** Nur Hamidah Abdul Halim

**Affiliations:** 1 Institute of Nano Electronic Engineering, Universiti Malaysia Perlis, Kangar, Malaysia

I would suggest the writer to include "non destructive/by *in situ* detection or measurement for fish freshness" in the abstract and title to highlight the novelty of this work.

 "The extracted ACN has a similarity over the FT-IR profile of cyanidin-3-glucoside" is suddenly introduced in the conclusion is hanging. Suggest elaborating how glycoside bonds are important and how it contributed to the pH changes or how it related to freshness. Then it can be concluded how the similarity by having FT-IR profile of cyanidin-3-glucoside in the ACN is desired in this work.

 Is having "cyanidin-3-glucoside in ACN" the actual highlight in this work? If yes, the novelty statement in the abstract, introduction and title need to be revised accordingly.

Is the work clearly and accurately presented and does it cite the current literature?

Yes

If applicable, is the statistical analysis and its interpretation appropriate?

Partly

Are all the source data underlying the results available to ensure full reproducibility?

Yes

Is the study design appropriate and is the work technically sound?

Partly

Are the conclusions drawn adequately supported by the results?

Yes

Are sufficient details of methods and analysis provided to allow replication by others?

Partly

Reviewer Expertise:

Electrochemical biosensors

I confirm that I have read this submission and believe that I have an appropriate level of expertise to confirm that it is of an acceptable scientific standard.

# Peer review: Reviewer response for version 1

**DOI:** 10.5256/f1000research.56160.r86281

**Published:** 2021-06-18

**Authors:** Nur Hamidah Abdul Halim

**Affiliations:** 1 Institute of Nano Electronic Engineering, Universiti Malaysia Perlis, Kangar, Malaysia

This paper shows an experimental work on optical sensor using simple optical approach. This paper shows a good work with a potential study on optical pH sensor for fish freshness monitoring. However, it is suggested to elaborate further on discussion how the mechanism and reaction with illustrated figure. The novelty should be explicitly mentioned in the introduction, abstract and findings. A table on reported or published and comparison should also be made available to see the research contribution. It is suggested that the authors may revise based on few comments below:

**Abstract**

*“The sensor displayed an excellent response after 10 minutes of exposure, possessing a response stability for 10 consecutive days. The decrease in pH value of the Tilapia fish from 7.3 to 5 was observed in a 48 hour test, which can be used as the parameter when monitoring fish freshness.“*

Comment: The statement of decrease in pH value need to be elaborated to highlight the novelty of this research. The authors may add comparison in terms of performance and mechanism that differentiate this works and other reported work. E.g How pH value decrease mechanism is evaluated and correlated to observe the fish freshness.

**Introduction**

 Para 2: *"Nevertheless, these aforementioned pH sensors could only be used on solutions with near-neutral pH as more basic or acidic solutions will give an insignificant response time. Pourjavaher et al. ^11^ has designed an optical pH sensor based on cellulose nanofibers with red cabbage (Brassica oleracea) extract, while Rajan et al. (2018) ^12^ has produced an optical pH sensor using peonidin pigment. However, this study did not report the working pH range of peonidin. The use of anthocyanin (ACN) from blackberries and chitosan membrane in an optical pH sensor has been established.13 The interaction and mechanical properties of chitosan membrane with entrapped ACN have also been reported."*

Comment: This paragraph should elaborate more on fish freshness and its correlation to pH based on previous study. The use of ACN should be illustrated for reader to understand more as the sentences is hanging (Referring to *“The interaction and mechanical properties of chitosan membrane with entrapped ACN have also been reported.”*). It is more helpful if a table or illustrated mechanism is shown to support this study and having a good flow of this paper.

 Para 3: *"A more recent study on fish freshness monitoring through optical methods was reported by Moradi et al. ^15^ using nanofiber bacterial cellulose with ACN. However, this method requires a relatively long analytical time as the pH measurement could not be conducted in situ. Chen et al. (2020) ^6^ has developed a sensitive novel film prepared from starch polyvinyl alcohol and starch polyvinyl alcohol glycerol."*

Comment: Again, this paragraph does not add the value on published work with this work. The mechanism on optical pH to monitor fish freshness is still not addressed. No comparison on the electrochemical performance (LOD, Linear range, selectivity) was mentioned here. How long analytical time is related to pH measurement by having different material like nanofiber and optical properties coming from ACN dye. The ACN sensitivities towards pH correlation to ACN optical properties may need to be added here as well.

**Research and Methodology**

Comment: The methodology shows a sufficient description a to give reader a good understanding on how this study is conducted. It is suggested that the authors may add process flow/illustration to complete the overall picture on steps and its mechanism.

**Results and Discussion**

 Figure 4. SEM profile of (a) PC and (b) ACN/PC membranes.

Comments: The morphology of ACN/PC membrane does not seem like a crack. It seems to have a wavy layer of membrane that might be the contributed to the adhesion/stress tension or air gap of the ACN/PC compared to PC alone. Is there any study on different ration of CAN added to this PC, or is it already optimized? The caption should be more detailed.

*"Color change of ACN can be affected by several factors such as temperature, pH, light intensity, sugar moiety and different phenolic derivatives. Due to its solubility in aqueous solution, the color change of ACN is caused by structural transformations of carbon skeleton affected by the levels of H+."*

 Comments: The color change mechanism is important to be introduced earlier in the introduction section and can be help with illustration. How different phenolic derivatives change this CAN, and which phenolic derivatives took place in this reaction? The authors may put or add this point to support the color change mechanism towards fish freshness from the finding.

**Effect of PC weight towards sensor sensitivity**

Comments: The pectin is a membrane that hold the ACN dye to improve the sensitivities. From Fig 4, the importance of having optimum load/weight of pectin is important the membrane with less surface tension, and this is the reason of having crack or wavy like membrane. It is very important optimum ratio of CAN/PC to have smooth ACN/PC membrane in this study.

Is the work clearly and accurately presented and does it cite the current literature?

Yes

If applicable, is the statistical analysis and its interpretation appropriate?

Partly

Are all the source data underlying the results available to ensure full reproducibility?

Yes

Is the study design appropriate and is the work technically sound?

Partly

Are the conclusions drawn adequately supported by the results?

Yes

Are sufficient details of methods and analysis provided to allow replication by others?

Partly

Reviewer Expertise:

Electrochemical biosensors

I confirm that I have read this submission and believe that I have an appropriate level of expertise to confirm that it is of an acceptable scientific standard, however I have significant reservations, as outlined above.

# Response

**Published:** 2021-07-09

**Authors:** Nazaruddin .

**Affiliations:** Universitas Syiah Kuala, Indonesia

Reviewer Nurhamidah

 Thank you very much for valuable comments

**Comment**: The statement of decrease in pH value need to be elaborated to highlight the novelty of this research. The authors may add comparison in terms of performance and mechanism that differentiate this works and other reported work. E.g How pH value decrease mechanism is evaluated and correlated to observe the fish freshness.

**Response**: The pH of fresh tilapia was 7.3, and the pH decreased to 5 after 7 hours of storage in two storage conditions (room temperature and 4oC). Changes in pH from 7.3 to 8.7 are the condition of fish monitoring within 48 hours. The post mortem glycolysis-derived lactic acid accumulation is also responsible for the pH decrease.

 Additionally, we have amended the manuscript with the following text:

 “ *Our method of measuring the change of pH is different to the most reported studies using colorimetric response. Indeed, one may argue that colorimetry could give the best practicality of the sensor use. However, it suffers from quantitative information, as it depends on the RGB profiles that requires complex model to convert the response into measured pH value. Moreover, the reported studies rely on the volatile basic compounds released from the meat. Taken altogether, the reported studies were unable to capture the decrease of pH during rigor mortis phase. In food industry, fish meat is best processed by the filleting machine during the pre- or post-rigor mortem. This is the novelty of our optical pH sensor which is useful for the quality control and processing of fish meat in industrial settings*.”

**Comment**: This paragraph should elaborate more on fish freshness and its correlation to pH based on previous study. The use of ACN should be illustrated for reader to understand more as the sentences is hanging (Referring to “The interaction and mechanical properties of chitosan membrane with entrapped ACN have also been reported.”). It is more helpful if a table or illustrated mechanism is shown to support this study and having a good flow of this paper.

**Response**: The paragraph 2 has been elaborated:

*Nevertheless, these aforementioned pH sensors could only be used on solutions with near-neutral pH as more basic or acidic solutions will give an insignificant response time. Pourjavaher et al. ^11^ has designed a pH sensor using bacterial cellulose (BC) nanofiber matrix to immobilize anthocyanin (CAN) from red cabbage (Brassica oleracea) extract. The sensor has a fairly wide pH range but it needs further characterization to evaluate the sensor performance, especially, for real foodstuff analysis. The use of ACN from blackberries and chitosan membrane in an optical pH sensor has been established. ^13^ The interaction and mechanical properties of chitosan membrane with entrapped ACN have also been reported. ^14^ Anthocyanins are flavonoids possessing a number of hydroxyl groups contributing a strong interaction with chitosan via hydrogen bonding.*

**Comment**: Again, this paragraph does not add the value on published work with this work. The mechanism on optical pH to monitor fish freshness is still not addressed. No comparison on the electrochemical performance (LOD, Linear range, selectivity) was mentioned here. How long analytical time is related to pH measurement by having different material like nanofiber and optical properties coming from ACN dye. The ACN sensitivities towards pH correlation to ACN optical properties may need to be added here as well.

**Response**: The paragraph 3 has been elaborated:

*A more recent study on fish freshness monitoring through optical methods was reported by Moradi et al. ^15^ using nanofiber bacterial cellulose with ACN. However, this method requires a relatively long analytical time as the pH measurement could not be conducted in situ. Chen et al. (2020) ^6^ has developed a sensitive novel film prepared from starch polyvinyl alcohol and starch polyvinyl alcohol glycerol. The study used curcumin from turmeric and anthocyanin from purple sweet potatoes. The results showed that the mixture of curcumin and ACN improved the stability than that of the individual active substances. As the consequence, the sensor could be employed to detect volatile ammonia as the fish freshness indicator.*

**Comment**: The methodology shows a sufficient description a to give reader a good understanding on how this study is conducted. It is suggested that the authors may add process flow/illustration to complete the overall picture on steps and its mechanism.

**Response**: The steps have been added Methods:

***Study Design***

*The first step in sensor fabrication was the extraction of anthocyanin from Ruellia tuberosa L. The extracted anthocyanins were then mixed with pectin solution and printed proportionally as an optical pH sensor. The optical pH sensor was then characterized and the optimized and then applied to monitor the freshness of tilapia. The image below is a schematic diagram summarizing research procedures conducted in this work.*

[Figure]

**Comment**: The morphology of ACN/PC membrane does not seem like a crack. It seems to have a wavy layer of membrane that might be the contributed to the adhesion/stress tension or air gap of the ACN/PC compared to PC alone. Is there any study on different ration of CAN added to this PC, or is it already optimized? The caption should be more detailed.

**Response**: The ratio of ACN has been optimized based on the sensitivity and R ^2^, see Table 1 and Figure 8. The description for SEM images analysis has been revised per suggestion.

**Comment**: The color change mechanism is important to be introduced earlier in the introduction section and can be help with illustration. How different phenolic derivatives change this CAN, and which phenolic derivatives took place in this reaction? The authors may put or add this point to support the color change mechanism towards fish freshness from the finding.

**Response**: The anthocyanin structure under different pHs has been added in the manuscript as suggested (see Figure 7).

[Figure]

**Comment**: The pectin is a membrane that hold the ACN dye to improve the sensitivities. From Fig 4, the importance of having optimum load/weight of pectin is important the membrane with less surface tension, and this is the reason of having crack or wavy like membrane. It is very important optimum ratio of CAN/PC to have smooth ACN/PC membrane in this study.

**Response**: We have optimized the PC weight and the optimum was reached for 0.1% PC to find optimum sensitivity. The membrane with 0.1% w/v pectin has a flatter surface thus making it as the most suitable optical sensor. SEM characterization was carried out on the optimum pectin weight. The wavy like surface structure was probably due to the addition of anthocyanin.

 Added as a recommendation in conclusion:

*More studies indeed need carried out to obtain smooth surface morphology to improve the optical sensor performance.*

 Due to the limited features in this comment column, we have uploaded our full response through this link.

# Peer review: Reviewer response for version 1

**DOI:** 10.5256/f1000research.56160.r86206

**Published:** 2021-06-04

**Authors:** Sagir Alva

**Affiliations:** 1 Department of Mechanical Engineering, Faculty of Engineering, Universitas Mercu Buana, Jakarta, Indonesia

After I read and reviewed this article, I found the theme of this article quite interesting. However, unfortunately, there are shortcomings in this article which make it unfit for indexing. Therefore, I suggest a Not Approval status for this article. However, it can be improved in the revision with the following comments:

 1. It is true, when fish begin to undergo a process of decomposition, in addition to producing H ^+^, ammonia is also produced. In the first paragraph, the authors only compared it with ammonia-ISE, where basically, the concept of measuring ammonia-ISE is an indirect measurement of ammonia based on the dissociation of ammonia in solution to form NH _4_^+^. So naturally not all ammonia will be detected. However, there are actually a lot of research on ammonia optical sensors. In other words, the ammonia optical sensor is nothing new. So there needs to be an explanation added to the introduction why choosing an optical pH sensor in detecting the freshness of fish compared to an ammonia optical sensor. What are the advantages of an optical pH sensor compared to an optical ammonia sensor?

 2. At the end of the first paragraph, you stated that measuring pH using an optical sensor might be good for samples that have interfering ions. With fish, what ions are supposed to be can interfere with the pH-ISE sensor, so you end up choosing the optical pH sensor over the pH-ISE sensor? An explanation of this needs to be added in the introduction section.

 3. Basically, a lot of plants and fruits also have ACN, and here you have also given examples such as blackberries. But why in this study have you focused on the *Ruellia* flower? Instead, you can also use ACN from blackberries immobilized using Pectin. What are the advantages of ACN from *Ruellia* compared to other plants? It is worth mentioning in the introduction the reasons for this.

 4. The use of a hydrogel membrane will indeed facilitate the diffusion of the analyte. However, the hydrogel membrane has serious problems such as easy to swell and break, so that the dye used can be leached and the sensor life time is decreased. There needs to be some clarification on this. In addition, there needs to be additional experimental data on the % swelling index of the pectin membrane used.

 5. In optical sensor, leaching study is an important thing to do. However, in this article there are no leaching study data, so it is necessary to add experimental data for leaching study testing.

 6. There are several natural hydrogel polymers. It is necessary to add reasons why choose Pectin over other natural hydrogel polymers. What are the advantages of pectin over other natural hydrogel polymers?

 7. On page 7 and the beginning of the first paragraph, there is the sentence: “The constructed optical pH biosensor based on the ACN derived from *R. tuberosa* L flower has hydrogel characteristics.” - It need clarification, is this really an optical biosensor? Because here I don't see any use of enzymes, peptides, micro-organisms etc.

 8. On page 7 it is stated that the colour change is caused by a structural transformation of the ACN. It is necessary to add pictures of the changes in the chemical structure of ACN at various pH variations, such as acidic, neutral and basic.

 9. Still from page 7, you stated that one of the factors that caused the change in ACN colour was caused by light. You need to clarify, how do you control the light intensity during the test period, so that the colour of the ACN remains stable and how long can the light change the colour of the ACN?

 10. In the ACN variation data, the resulting absorbance will also decrease with the lower ACN concentration, and in the end you use a concentration of 0.025 mg/L as the optimum concentration of ACN. What if the concentration of the ACN is less than 0.025 mg/L? Is ACN still able to respond to changes in pH or not able to respond to changes in pH? Additional data are needed for testing less than 0.025 mg/L.

 11. In sensor development, validation testing is very important to ensure that the fabricated sensor performs at least the same as standard test equipment. Here, I don't see that. There needs to be additional validation data with standard methods to test the freshness of fish based on pH changes.

Is the work clearly and accurately presented and does it cite the current literature?

Partly

If applicable, is the statistical analysis and its interpretation appropriate?

Yes

Are all the source data underlying the results available to ensure full reproducibility?

Yes

Is the study design appropriate and is the work technically sound?

No

Are the conclusions drawn adequately supported by the results?

Yes

Are sufficient details of methods and analysis provided to allow replication by others?

Yes

Reviewer Expertise:

My specialty is the synthesis and characterization of materials such as polymers for the development of chemical sensors/biosensors.

I confirm that I have read this submission and believe that I have an appropriate level of expertise to state that I do not consider it to be of an acceptable scientific standard, for reasons outlined above.

# Response

**Published:** 2021-07-09

**Authors:** Nazaruddin .

**Affiliations:** Universitas Syiah Kuala, Indonesia

**Comment:** It is true, when fish begin to undergo a process of decomposition, in addition to producing H ^+^, ammonia is also produced. In the first paragraph, the authors only compared it with ammonia-ISE, where basically, the concept of measuring ammonia-ISE is an indirect measurement of ammonia based on the dissociation of ammonia in solution to form NH ^4+^. So naturally not all ammonia will be detected. However, there are actually a lot of research on ammonia optical sensors. In other words, the ammonia optical sensor is nothing new. So there needs to be an explanation added to the introduction why choosing an optical pH sensor in detecting the freshness of fish compared to an ammonia optical sensor. What are the advantages of an optical pH sensor compared to an optical ammonia sensor?

**Response: **

- The literature on the development of the NH3 optical biosensor was developed by Dan-Feng Lu and Zhi-mei Qi in 2019 using bromothymol blue and a porous glass membrane. This sensor can only work at low concentrations of ammonia.
- Another ammonia sensor has also been developed by Maximilian Maierhofer *et al.* (2020), who fabricated the sensor using fluorescence properties of aza-BODIPY dyes with a response time of 390 seconds.
- Detection of ammonia as a total volatile basic nitrogen (TVB-N) to determine fish spoilage requires a sample destruction process (Nathan Wells *et al.*(2019), Talanta 194: 830–836). Then, the standard curve was obtained from measuring the absorbance of the trimethylamine (TMA) compound that produces ammonia through a complicated procedure. On the other hand,  this method was also based on pH measurements. So it can be concluded that ammonia is also correlated with changes in pH to determine the freshness of fish. The same concept has also been previously reported by T. Werner *et al.* (1995) Analyst 120 1627–1631 where the determination of ammonia was based on measuring pH using an ion-pair indicator. Therefore, the detection of fish freshness through pH measurements is more representative of the actual condition of *in-situ *tests.
- An explanation of the ammonia optical sensor and its drawbacks for determining fish freshness has been described in the introduction.

**Comment:** At the end of the first paragraph, you stated that measuring pH using an optical sensor might be good for samples that have interfering ions. With fish, what ions are supposed to be can interfere with the pH-ISE sensor, so you end up choosing the optical pH sensor over the pH-ISE sensor? An explanation of this needs to be added in the introduction section.

**Response: **Literature reported ISE H ^+^ response is strongly affected by alkaline ions has been added in the Introduction.

**Comment: **Basically, a lot of plants and fruits also have ACN, and here you have also given examples such as blackberries. But why in this study have you focused on the Ruellia flower? Instead, you can also use ACN from blackberries immobilized using Pectin. What are the advantages of ACN from Ruellia compared to other plants? It is worth mentioning in the introduction the reasons for this.

**Response**: Mostly, coloured plants contain anthocyanins, including blackberries. Anthocyanins from blackberries can also be used as pH-sensitive active ingredients to develop optical pH sensors. On the other hand, sources of anthocyanins from blackberries are difficult to obtain in our area. In this study, *Ruellia* anthocyanins were used as a sensitive pH compound for optical pH sensor development because the flowers are easy to obtain. In addition, based on a preliminary study on the sensitivity of the anthocyanin at various pHs, we found that the anthocyanin has a great potential to be further applied in developing optical pH sensor.

**Comment: **The use of a hydrogel membrane will indeed facilitate the diffusion of the analyte. However, the hydrogel membrane has serious problems such as easy to swell and break, so that the dye used can be leached and the sensor life time is decreased. There needs to be some clarification on this. In addition, there needs to be additional experimental data on the % swelling index of the pectin membrane used.

**Response**: Firstly, the membrane use as optical pH sensor is not applied by immersion into aqueous samples therefore swelling index is not relevant. Secondly, there have been extensive research pertaining to the swelling profile of pectin, of which are Fong H. WEH *et al.* (2014) Lat. Am. J. Pharm. 33(3): 420-31 and  Naziha Chirani *et al.* 2015. Journal of Biomedical Sciences. Vol. 4 No. 2:13. P 1-23.

 Below is swelling index of pectin in different media based on the reported study.

[Figure]

**Comment: **In optical sensor, leaching study is an important thing to do. However, in this article there are no leaching study data, so it is necessary to add experimental data for leaching study testing.

**Response: **In our opinion, not all leaching tests need to be carried out in sensor or biosensor manufacturing studies. It depends on the sensor application. In this study, we did not immerse the sensor in the sample. The sensor is placed directly on the surface of the fish, and then the colour changes are measured. For a liquid sample, only a small amount of sample dropped onto the sensor surface. The sensor produced is a disposal sensor.

**Comment**: There are several natural hydrogel polymers. It is necessary to add reasons why choose Pectin over other natural hydrogel polymers. What are the advantages of pectin over other natural hydrogel polymers?

**Response: **Pectin was chosen because of:

1. Its non-toxicity; because the application is for a foodstuff, the sensor should not be toxic.
2. Its ability in forming membrane structure.
3. Transparent and homogenous.
4. In the case of optical pH sensor for fish freshness monitoring, other studies have reported chitosan, starch, and cellulosic materials; while pectin is scarcely reported. Hence, the use of pectine is a novelty.

Those characteristics have been added in the last paragraph of introduction.

**Comment**: On page 7 and the beginning of the first paragraph, there is the sentence: “The constructed optical pH biosensor based on the ACN derived from *R. tuberosa* L flower has hydrogel characteristics.” - It need clarification, is this really an optical biosensor? Because here I don't see any use of enzymes, peptides, micro-organisms etc.

**Response**: It is a sensor not as a biosensor. Has been modified: “biosensor”  to “sensor”

**Comment**: On page 7 it is stated that the colour change is caused by a structural transformation of the ACN. It is necessary to add pictures of the changes in the chemical structure of ACN at various pH variations, such as acidic, neutral and basic.

**Response**: Has been added, see figure 7 [Figure].

**Comment**: Still from page 7, you stated that one of the factors that caused the change in ACN colour was caused by light. You need to clarify, how do you control the light intensity during the test period, so that the colour of the ACN remains stable and how long can the light change the colour of the ACN?

**Response**: The sensor has been made through a storage process in a dark condition and a temperature of 4 ^o^C. At the time of measurement, the sensor is also kept in the dark and needs a short time of exposure to light during the measurement process. We predict no significant colour change. In addition from our preliminary experiment, immobilized anthocyanins on the pectin matrix have good stability.

**Comment**: In the ACN variation data, the resulting absorbance will also decrease with the lower ACN concentration, and in the end you use a concentration of 0.025 mg/L as the optimum concentration of ACN. What if the concentration of the ACN is less than 0.025 mg/L? Is ACN still able to respond to changes in pH or not able to respond to changes in pH? Additional data are needed for testing less than 0.025 mg/L.

**Response**: The effect of anthocyanin concentration is not significantly different on sensor sensitivity and linear range. Anthocyanin concentrations less than 0.025 mg/L are predicted still to respond to pH changes. Due to the intensity of the colour decreases, the sensitivity will also decrease. Thus, the determination of sensitivity for anthocyanin concentrations lower than 0.025 mg/L was not determined.

**Comment**: In sensor development, validation testing is very important to ensure that the fabricated sensor performs at least the same as standard test equipment. Here, I don't see that. There needs to be additional validation data with standard methods to test the freshness of fish based on pH changes.

**Response**: We have validated the optical sensor method using H ^+^ ion-selective electrodes. However, we do not report it. In this paper, we focus more on how the pH changes in fish stored at room temperature and 4 ^o^C. The following are the results of the validation of measurements carried out on fish measured using an optical pH sensor with H ^+^ ISE.

[Table]

 From the results obtained that the ISE measurement is influenced by temperature (as also suggested by other reported studies) so that the results obtained are different from the optical pH sensor.

 Due to the limited features available in this comment column, we choose to upload our full response in an accessible link. Please find it through this link.
